# Non-additive genetic variation in growth, carcass and fertility traits of beef cattle

**DOI:** 10.1186/s12711-015-0114-8

**Published:** 2015-04-02

**Authors:** Sunduimijid Bolormaa, Jennie E Pryce, Yuandan Zhang, Antonio Reverter, William Barendse, Ben J Hayes, Michael E Goddard

**Affiliations:** Victorian Department of Economic Development, Jobs, Transport and Resources, Bundoora, VIC 3083 Australia; School of Land and Environment, University of Melbourne, Parkville, VIC 3010 Australia; Animal Genetics and Breeding Unit, UNE, Armidale, NSW 2351 Australia; CSIRO Animal, Food and Health Sciences, Queensland Bioscience Precinct, St. Lucia, QLD 4067 Australia

## Abstract

**Background:**

A better understanding of non-additive variance could lead to increased knowledge on the genetic control and physiology of quantitative traits, and to improved prediction of the genetic value and phenotype of individuals. Genome-wide panels of single nucleotide polymorphisms (SNPs) have been mainly used to map additive effects for quantitative traits, but they can also be used to investigate non-additive effects. We estimated dominance and epistatic effects of SNPs on various traits in beef cattle and the variance explained by dominance, and quantified the increase in accuracy of phenotype prediction by including dominance deviations in its estimation.

**Methods:**

Genotype data (729 068 real or imputed SNPs) and phenotypes on up to 16 traits of 10 191 individuals from *Bos taurus*, *Bos indicus* and composite breeds were used. A genome-wide association study was performed by fitting the additive and dominance effects of single SNPs. The dominance variance was estimated by fitting a dominance relationship matrix constructed from the 729 068 SNPs. The accuracy of predicted phenotypic values was evaluated by best linear unbiased prediction using the additive and dominance relationship matrices. Epistatic interactions (additive × additive) were tested between each of the 28 SNPs that are known to have additive effects on multiple traits, and each of the other remaining 729 067 SNPs.

**Results:**

The number of significant dominance effects was greater than expected by chance and most of them were in the direction that is presumed to increase fitness and in the opposite direction to inbreeding depression. Estimates of dominance variance explained by SNPs varied widely between traits, but had large standard errors. The median dominance variance across the 16 traits was equal to 5% of the phenotypic variance. Including a dominance deviation in the prediction did not significantly increase its accuracy for any of the phenotypes. The number of additive × additive epistatic effects that were statistically significant was greater than expected by chance.

**Conclusions:**

Significant dominance and epistatic effects occur for growth, carcass and fertility traits in beef cattle but they are difficult to estimate precisely and including them in phenotype prediction does not increase its accuracy.

## Background

Mutations of large effects often show non-additive effects on the phenotype such as dominance and epistasis and one well-known example is the coat colour of mice [1]. However, it is uncertain how important these non-additive effects are for polymorphisms that control variation in complex or quantitative traits. Hill et al. [2] argued that even if gene effects are not additive, most of the genetic variance is still expected to be additive variance. Several studies have estimated epistatic or dominance variance in livestock using traditional pedigree information (e.g., [3-7]) and reported a small but significant non-additive variance. However, it is difficult to estimate non-additive variance because it is often, at least partially, confounded with other effects such as common environment or maternal effects. Consequently, estimates of non-additive variance may be biased upwards. In view of this, it is not surprising that most genetic evaluation systems use an additive model and ignore non-additive effects, especially considering that their aim is to estimate breeding values or additive genetic values.

Genome-wide dense single nucleotide polymorphisms (SNPs) have been widely used in cattle for association studies [8-12] and genomic prediction [13-15] and represent a new opportunity to estimate non-additive effects at individual loci and to estimate non-additive variances. However, most genome-wide association studies (GWAS) have only reported additive effects of SNPs and additive genetic variance. There are a few studies (e.g., [6]) based on high-density SNPs that have reported variance explained by non-additive effects. In a study on growth rate (daily gain) in pigs, Su et al. [6] estimated dominance variance and additive × additive epistatic variance to account for 5.6 and 9.5% of the total phenotypic variance, respectively. It is possible that the proportion of variance that is explained by non-additive effects varies between traits.

Therefore, the objective of this study was to estimate dominance and epistatic effects in beef cattle using high-density SNP genotypes and phenotypic data on growth, carcass and reproductive traits.

## Methods

### Animals, phenotypes and SNP data

Animals originated from nine different populations of three breed types. They included four *Bos taurus* breeds (Angus, Murray Grey, Shorthorn, Hereford), one *Bos indicus* breed (Brahman cattle), three composite breeds (Belmont Red, Santa Gertrudis, Tropical composites), and one recent Brahman cross population (F1 crosses of Brahman with Limousin, Charolais, Angus, Shorthorn, and Hereford). The structure of the populations used is described in Bolormaa et al. [15]. A total of 10 191 animals of the three breed types (3389 *B. indicus*, 3296 *B. taurus*, and 3506 *B. taurus* × *B. indicus*) with SNP genotypes and phenotypes for at least one trait were used.

Phenotypes for 16 different traits (Table 1), including growth, feed intake, carcass and meat quality and fertility traits were obtained from five sources, which will be referred to as datasets: the Beef Cooperative Research Centre Phase I (CRCI), Phase II (CRCII), Phase III (CRCIII), the Trangie selection lines, and the Durham Shorthorn group (for a detailed description, see [15-17]). All individuals were not measured for all traits. The number of genotyped cattle with each trait in each dataset, trait definitions and abbreviations, means and standard deviations (SD), and heritability estimates are in Table 1 and were obtained from Bolormaa et al. [15,18]. The data for two female fertility traits (age at first detected corpus luteum and post-partum anoestrus interval PPAI) used in this study were adjusted for non-genetic effects based on models described in previous studies [19,20].

**Table 1:** **Number of genotyped animals and number of phenotypes, and mean, standard deviation (SD) and heritability estimates (h** ^**2**^ **) of each trait for animals with a full set of records** ^**1**^

**Trait** ^**2**^	**Nb** ^**3**^	**Total**	**Mean**	**SD**	**h** ^**2**^	**Trait name**
PW_hip	6359	10515	119.0	7.9	0.53	Hip height measured post weaning (cm)
X_hip	2037	4730	138.8	7.7	0.45	HH measured at feedlot exit (cm)
HUMP	1132	2099	140.4	37.1	0.29	Hump height as assessed by MSA grader (mm)
PW_lwt	9884	16079	230.9	53.4	0.45	Live weight measured post weaning (kg)
X_lwt	5992	11599	497.9	97.9	0.42	Live weight measured at feedlot exit (kg)
RFI	4026	4837	−1.5	2.1	0.36	Residual feed intake (kg)
PWIGF	918	1678	262.2	147.4	0.25	IGF-I concentration measured post weaning (ng/ml)
CP8	5727	11061	11.3	5.0	0.35	Fat depth at P8 site (mm)
CRIB	5464	10690	7.4	4.1	0.31	Fat depth at rib site (mm)
CIMF	5824	11200	3.4	1.9	0.40	Intra-muscular fat (%)
CRBY	2684	3639	66.9	3.4	0.47	Carcass retail beef yield (%)
LLPF	5358	10327	4.5	1.0	0.25	Peak force measured in *longissimus dorsi* muscle (kg)
SC12	1112	1112	21.2	2.7	0.62	Scrotal circumference measured at ages of 12 months (cm)
PNS24	964	964	73.6	22.1	0.23	Percentage of normal sperm at the age of 24 months (%)
AGECL	2045	2057	698.7	140.4	0.52	Age at first detected corpus luteum (days)
PPAI	1448	1455	158.2	110.8	0.49	Post partum anoestrus interval (days)

^1^This summary statistics for the above traits can be found in Bolormaa et al. [15,16]; ^2^trait abbreviation; ^3^number of genotyped animals.

Data on 729 068 SNPs were imputed from genotypes from five different SNP panels: (1) the Illumina HD Bovine SNP chip (http://res.illumina.com/documents/products/datasheets/datasheet_bovinehd.pdf) that contains 777 963 SNPs; the BovineSNP50K BeadChip (Illumina, San Diego) version 1 (2) and version 2 (3) that contain 54 001 and 54 609 SNPs, respectively; (4) the Illumina SNP7K panel that comprises 6909 SNPs; and (5) the Parallele SNP10K chip (Affymetrix, Santa Clara, CA) with 11 932 SNPs. All SNPs were mapped to the UMD 3.1 assembly of the bovine genome sequence provided by the Centre for Bioinformatics and Computational Biology at University of Maryland (CBCB) (ftp://ftp.cbcb.umd.edu/pub/data/assembly/Bos_taurus). Procedures for stringent quality control of the SNP data and for imputation to 729 068 SNPs are described in Bolormaa et al. [15]. The genotypes for each SNP were encoded in the top/top Illumina A/B format (http://res.illumina.com/documents/products/technotes/technote_topbot.pdf).

### Statistical analyses

#### Genome-wide association studies by fitting dominance

*Model used for GWAS*: GWAS was performed using the ASReml software [21] based on the following mixed model:

1 $$ \mathbf{y}={\mathbf{1}}_{\mathbf{n}}\mu +\mathbf{X}\mathbf{b}+{\mathbf{s}}_{\mathbf{i}}{\alpha}_i+{\mathbf{w}}_{\mathbf{i}}{\delta}_i+\mathbf{Z}\mathbf{a}+\mathbf{e}, $$

where **y** is the vector of observed phenotypic values of the animals, **1**_**n**_ is an nx1 vector of 1′s (n is the number of animals with phenotypes), μ is the overall mean, **b** is a vector of fixed effects, **X** is a design matrix relating observations to the corresponding fixed effects, **Z** is a design matrix relating observations to random animal genetic effects, **a** is a vector of polygenic breeding values sampled from N ~ (0, **A**σ_a_^2^), where σ_a_^2^ is additive genetic variance and **A** is the additive relationship matrix constructed from the pedigree of the animals and their 5-generation-ancestors; **s**_**i**_ is a vector of additive genotype codes of each animal at the *i*-th SNP, with genotypes AA, AB and BB coded as 0, 1 or 2, respectively, i.e. according to the number of B alleles present, α_i_ is the additive effect of the *i*-th SNP, **w**_**i**_ is a vector of dominance genotype codes at SNP_i_ with the heterozygote AB coded as 1 and the homozygotes AA and BB coded as 0, δ_i_ is the dominance effect of *i-*th SNP, and **e** is the vector of random residual effects. Vectors **s**_**i**_ and **w**_**i**_ were fitted as covariates. In this parameterization, significance of the dominance effect was tested after fitting the additive effect. This analysis was carried out once for each SNP, i.e. 729 068 times.

The model included dataset, breed, cohort and sex as fixed effects for all traits, except for the two female fertility traits. Other fixed effects varied by trait, as detailed in [19,22-26]. The fixed effects were fitted as nested within a dataset.

Following Bolormaa et al. [27], the false discovery rate (FDR) of SNP effects was estimated as:

$$ \frac{P\left(1-\frac{A}{T}\right)}{\left(\frac{A}{T}\right)\left(1-P\right)}, $$

where *P* is the *P*-value used to declare the additive or dominance effect of a SNP to be significant (e.g., 0.00001), *A* is the number of SNP effects that were declared significant at the stated *P* -value and *T* is the total number of SNPs tested. Although the true FDR cannot exceed 100%, the estimated FDR can exceed 100% if the number of significant SNPs is smaller than expected by chance, but the interpretation is simply that all SNPs discovered are false positives.

### Validation of SNPs with dominance effects

In order to validate statistically significant SNP effects in an independent population, the animals in the complete dataset were split into five sets by allocating the offspring of randomly selected sires to one of the five datasets. Then, one of the five sets was used as a validation population and the four other sets as the reference population. In this way, no animal used for validation had paternal half-sibs in the reference population. GWAS for each trait was performed in the reference population. For SNPs with a significant dominance effect in the reference population, the analysis was repeated in the validation population. We counted the number of times that the direction of the estimated SNP effect was the same in the validation and discovery populations.

### Dominance variance explained by SNP genotypes

The dominance variance was estimated by restricted maximum likelihood (REML) using the following model:

2 $$ \mathbf{y}={\mathbf{1}}_{\mathbf{n}}\mu + \mathbf{X}\mathbf{b}+\mathbf{h}\mathbf{e}\mathbf{t}\mathrm{\ss } + \mathbf{g}+\mathbf{d}+\mathbf{e}, $$

where **y** is the vector of observed phenotypic values of the animals, **1**_**n**_ is a vector of 1′s, μ is the overall mean, **X** is a design matrix relating observations to the corresponding fixed effects where the fixed effects were the same as used in the GWAS, **b** is vector of fixed effects, **het** is a vector containing the average heterozygosity over all SNPs for each animal, ß is the regression of each trait on heterozygosity, **g** is a vector of genomic breeding values distributed as N ~ (0, **G**σ_g_^2^), where σ_g_^2^ is additive genetic variance explained by SNPs and **G** is the genomic relationship matrix (GRM) [28], **d** is a vector of dominance deviations distributed as N ~ (0, **D**σ_d_^2^), where σ_d_^2^ is dominance variance explained by the SNPs and **D** is the dominance relationship matrix (DRM), and **e** is the vector of random residual effects. The GRM was constructed using the genotypes of all 10 191 animals in the combined datasets according to [28]. More details on the GRM used in this study are in [15]. The DRM was derived as described below.

Let the allele frequency of A be q and that of B be p = 1-q. The genotype of the *j-*th animal at the *i-*th SNP (AA, AB, and BB) was coded as H_ij_ = −p/q, +1, and –q/p, respectively. Then, in a base population in Hardy-Weinberg equilibrium:

$$ E\left({H}_{ij}\right)=-\frac{p}{q}{q}^2+2pq-\frac{q}{p}{p}^2=0 $$

and

$$ E\left({H}_{ij}^2\right)=\frac{p^2}{q^2}{q}^2+2pq+\frac{q^2}{p^2}{p}^2=1\ . $$

The additive contribution of the genotype of the *j-*th animal (T_ij_) is coded as -2p/ $\sqrt{2pq}$, (q-p)/ $\sqrt{2pq}$, and 2q/ $\sqrt{2pq}$ for AA, BB, and BB genotypes, respectively. Then, the expectation of the covariance between additive and dominance contributions to the genotype is as follows:

$$ \begin{array}{l}E\left({H}_{ij}{T}_{ij}\right)=\frac{-\frac{p}{q}\left(-2p\right){q}^2+1\left(q-p\right)2pq-\frac{q}{p}2q{p}^2}{\sqrt{2pq}}\hfill \\ {}=\frac{2{p}^2q+2p{q}^2-2{p}^2q-2p{q}^2}{\sqrt{2pq}}=0.\hfill \end{array} $$

This shows that allele substitution effects and dominance deviations that are implicit in the model are orthogonal contrasts. Therefore, DRM = **HH’**/m and GRM = **TT’**/m, where m is the total number of SNPs, **H** is a matrix of dimension of the number of animals x number of SNPs, where H_ij_ is present at each position in the matrix; in the same way, **T** is a matrix containing T_ij_. With this model, σ_d_^2^ is the classical definition of dominance variance. Model (2) is equivalent to a model in which **g** is replaced by **Tu**, Var(**u**) = **I**σ_a_^2^/m, and **d** is replaced by **Hv**, Var(**v**) = **I**σ_d_^2^/m where **u** and **v** are vectors containing the additive and dominant effects of each SNP.

This definition of the DRM is similar to that used by Vitezica et al. [29], except that in our study, the contributions of each SNP to the DRM was weighted by 1/(2pq). In the analysis of each trait, only the elements of the GRM and DRM corresponding to animals with phenotypes were used to reduce the computing time. The same fixed effects as those used in the GWAS (model 1) were fitted in this model. The estimates of variance components were performed using ASReml software [21].

The significance of the dominance variance was tested by comparing twice the difference in log-likelihood between additive genomic model (AM) and additive and dominance genomic model (ADM) to a chi-squared distribution with degrees of freedom of 1.

### Accuracy of predictions of the phenotypic values by including dominance deviations

For validation purposes, predictions of additive and dominance genetic values were made using the best linear unbiased prediction (GBLUP) analysis with variance estimated by REML. A five-fold cross validation scheme was carried out. The data were split into five parts of approximately equal size, by allocating the offspring of each sire to one of the five datasets. In this way no animal used for validation had paternal half sibs in the discovery population. The analysis was performed five times using each dataset in turn as a validation group and the four other sets as the discovery population. Validation animals were included in the GRM and DRM but had missing phenotypes in the calculation of genomic estimated breeding values (GEBV). For each validation population, the genetic and phenotypic value of each animal in the validation population was predicted as $\widehat{\boldsymbol{g}}=\widehat{\boldsymbol{a}}+\widehat{\boldsymbol{d}}$, where ***ĝ*** is a predicted total genetic value, ***â*** is the estimated genomic breeding value, and $\widehat{\boldsymbol{d}}$ is the estimated dominance deviation predicted from ADM, that is model (2) above. Estimates of ***ĝ*** were obtained using ASReml software [21]. The phenotypic values for each trait were corrected for all fixed effects as: corrected phenotype = phenotype - fixed effects.

The accuracy of this prediction was calculated as the correlation between ***ĝ*** and the corrected phenotype within each breed. Accuracies were reported only when the number of records per breed was greater than 200. When combining accuracies across breeds, the overall accuracy was the mean accuracy within breeds weighted by the number of records of each breed. The accuracy when including dominance effects was compared to the accuracy obtained using the same model but without the dominance effects.

### Epistatic interactions between SNPs

Bolormaa et al. [18] identified 28 SNPs, referred to as lead SNPs, that had significant additive effects (*P* < 10^−5^) in a multi-trait analysis of the same traits as those used here. To minimise multiple-testing, we tested only additive × additive interactions between each of the 28 lead SNPs and all other 729 068 SNPs. Therefore, for each trait and for each lead SNP, we performed a GWAS in which we fitted the same model as for the dominance GWAS but, instead of a dominance effect, we fitted the additive effects of the lead SNP and one other SNP and the interaction between the two of them. The statistical model used for each trait was:

$$ \mathbf{y}={\mathbf{1}}_{\mathbf{n}}'\mu +\mathbf{X}\mathbf{b}+{\mathbf{l}}_{\mathbf{j}}{u}_j+{\mathbf{s}}_{\mathbf{i}}{a}_i + \left({\mathbf{l}}_{\mathbf{j}}\times {\mathbf{s}}_{\mathbf{i}}\right){m}_{ji}+\mathbf{Z}\mathbf{a}+\mathbf{e}, $$

where **y** is the vector of observed phenotypic values of the animals, **1**_**n**_ is a vector of 1′s, μ is the overall mean, **b** is a vector of fixed effects, **X** is a design matrix relating observations to the corresponding fixed effects, **Z** is a design matrix relating observations to the random animal effect, **a** is the vector of polygenic breeding values, **e** is the vector of random residual effect, **l**_**j**_ is a vector containing the coded genotypes for the *j-*th lead SNP (l_j_, j = 1, 2, 3, …, 28), u_j_ is the additive effect of the *j*-th lead SNP, **s**_**i**_ is a vector containing coded genotypes for the *i*-th SNP (SNP_i_, i = 1, 2, 3, … , 729 068), a_i_ is the additive effect of the *i-*th SNP, **l**_**j**_ **× s**_**i**_ is a vector of the interactions between the lead SNP_j_ and SNP_i_, and m_ji_ is the effect of the interaction between the lead SNP_j_ and SNP_i_. The effects of the *j-*th lead SNP, the *i-*th SNP, and their interaction (**l**_**j**_ **× s**_**i**_) were fitted simultaneously as covariates.

## Results

### Genome-wide association studies fitting dominance

**Figure 1:**
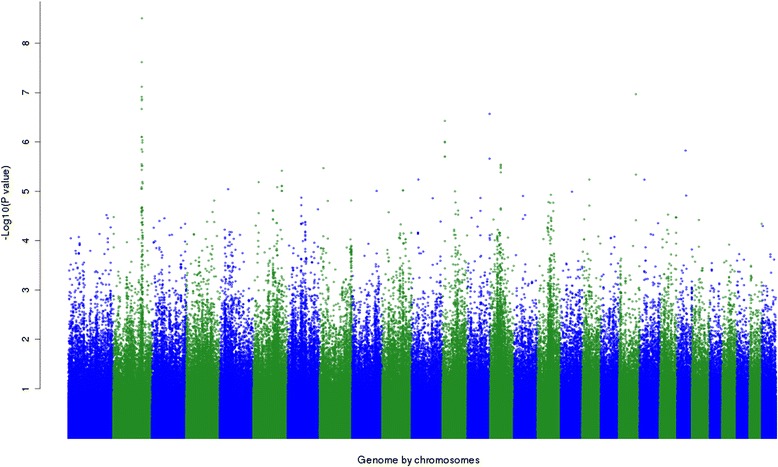
**Manhattan plot of the –log**_**10**_ **(** ***P*** **-values) of SNP dominance of the whole genome except chromosome X for live weight measured at feedlot exit (X_lwt).**

A Manhattan plot of the –log_10_(*P*-values) of SNP dominance effects is in Figure 1. The number of SNPs showing a significant (*P* < 10^−4^) dominance effect in the discovery population for each trait is in Table 2. For instance, for post-weaning hip height (PW_hip), 191 SNPs had significant (*P* < 10^−4^) dominance effects. Since 729 068 SNPs were tested, 70 significant SNPs are expected by chance alone and therefore the FDR was 36%. Among these 191 SNPs, 174 had a positive and 17 a negative dominance effect. In the validation population, only 185 of the 191 SNPs could be tested for dominance because, for the other six SNPs, not all three genotypes were represented in the validation population. Among the SNPs that had a positive effect in the discovery population, 66% also had a positive effect in the validation population and among those that had a negative effect in the discovery population, 56% also had a negative effect in the validation population. If the significant SNPs in the discovery population were all false positives, we would expect 50% of the effects to be in the same direction in the validation population. Based on a chi-squared test, 66% differs significantly (*P* < 0.001) from 50% but 56% does not differ significantly from 50%. For the trait, weight measured at feedlot exit (X_lwt), similar results were observed (Table 2).

**Table 2:** **Total number of SNPs with significant (** ***P*** **< 10** ^**−4**^ **) dominance effects, number of SNPs with positive (+ve) and negative (−ve) dominance effects in the discovery population, number of SNPs tested for validation of dominance effects (Nb tested), and percentage of SNPs with positive and negative dominance effects that had an effect in the same direction in the validation population for each trait**

**Trait** ^**1**^	**Total Nb**	**FDR (%)**	**+ve Nb**	**-ve Nb**	**Nb tested**	**+ve validated** ^**2**^ **(%)**	**-ve validated** ^**2**^ **(%)**
PW_hip	191	36.3	174	17	185	66^***^	56
X_hip	203	34.1	197	6	113	61^*^	25
HUMP	71	97.5	29	42	68	39	63
PW_lwt	207	33.4	197	10	205	74^***^	70
X_lwt	265	26.1	253	12	265	60^**^	67
RFI	106	65.3	44	62	105	57	89^***^
PWIGF	183	37.8	99	84	108	64	33^**^
CP8	63	109.9	24	39	60	54	50
CRIB	75	92.3	32	43	72	48	71^**^
CIMF	197	35.1	71	126	194	69^**^	61^*^
CRBY	114	67	56	58	112	38	41
LLPF	102	67.9	66	36	101	47	43
SC12	186	37.2	26	160	70	30	42
PNS24	260	26.6	249	11	80	51^*^	18^*^
AGECL	136	59	20	116	82	40	55
PPAI	90	76.9	27	63	67	58^*^	36^*^

^1^Trait abbreviations are in Table 1; ^2^percentage of significant SNPs (%) that had an effect in the same direction in the validation population: ^*^ those that significantly differ from the expected number of SNPs at *P* < 0.05; ^**^those that significantly differ from the expected number of SNPs at *P* < 0.01; and ^***^those that significantly differ from the expected number of SNPs at *P* < 0.001.

Table 2 shows that for height and weight traits and for percentage of normal sperm in semen, the number of positive dominance effects (d > 0) was greater than the number of negative dominance effects (d < 0). For residual feed intake, age at puberty and postpartum anoestrus interval, there were more negative effects. These results show dominance in the direction one might associate with increased fitness and in the direction of heterosis in crosses between breeds. In fact, the number of significant dominance effects in the direction expected to decrease fitness for these traits was smaller than expected by chance, while the number of effects in the expected direction (corresponding to heterozygote advantage) was greater than expected by chance for all these traits. We did not attempt to validate individual SNP dominance effects because the validation population was not large enough to have sufficient power for this. Instead, we attempted to validate the dominance effects as a group by observing the percentage of effects that had the same direction in the validation population as in the discovery population. For 11 of the 16 traits, the percentage of effects in the same direction was larger than 50%. The most convincing results were obtained for traits that show more dominance effects in the direction that is expected for heterosis and for which statistical power was greatest, i.e. height and weight traits. For other traits, such as fat depth, the FDR was high, the proportion of positive and negative effects was similar and the percentage of effects that had the same direction in the training and validation populations was close to 50%, which suggests that most of these effects were false positives. One exception was marbling measured by CIMF, for which the FDR was only 35%, the number of negative effects was greater and more than 50% of the SNPs had effects in the same direction in the training and validation populations.

### Dominance variance

Estimates of genetic variance using the additive genomic model (AM) and the model fitting both additive and dominance genetic effects (additive-dominance genomic model, ADM) are in Table 3 for each trait. The dominance variance was significant (*P* < 0.05) for PW_hip, PW_lwt, X_lwt, PWIGF, CIMF, CRBY and AGECL (Table 3). Estimates of the dominance variance as a proportion of phenotypic variance (V_D_/V_P_) varied from 0 to 0.42 but the standard errors were large in some cases (e.g., SC12 and PNS24). For seven traits, V_D_/V_P_ was estimated to be 0. Therefore, the average V_D_/V_P_ across traits was biased upwards but the median, which was 0.05, should not be biased. The only fertility trait that had a significant dominance variance was AGECL (18%). Dominance variance was not significant for both male fertility traits (SC12 and PNS24) and for PPAI (Table 3), which may be due to the low number of records for these traits (Table 2), large environmental variance and low relationships among recorded animals, which results in lack of power to estimate dominance variance.

**Table 3:** **Proportion of genetic variance based on the additive genomic model (AM) and the additive and dominance genomic model (ADM) for each trait**

	**AM**	**ADM**					
**Trait** ^**1**^	**V**_**A**_ **/V**_**P**_	**V**_**P**_^**(2)**^	**t**_**He**_	**V**_**A**_ **/V**_**P**_	**V**_**D**_ **/V**_**P**_	**V**_**G**_ **/V**_**P**_	**V**_**D**_ **/V**_**G**_ **(%)**
PW_hip	0.57 (0.03)	15.9	7.53^***^	0.57 (0.03)	0.04 (0.02)^*^	0.62 (0.03)	7
X_hip	0.47 (0.06)	22.0	2.77^**^	0.47 (0.06)	0 .00 (0.00)	0.47 (0.06)	0
HUMP	0.29 (0.08)	584.3	−4.15^***^	0.29 (0.08)	0.00 (0.00)	0.29 (0.08)	0
PW_lwt	0.39 (0.02)	547.1	7.19^***^	0.39 (0.02)	0.11 (0.02)^***^	0.50 (0.03)	23
X_lwt	0.46 (0.03)	2056.0	7.46^***^	0.47 (0.03)	0.07 (0.03)^**^	0.54 (0.04)	13
RFI	0.43 (0.04)	0.9	0.11	0.43 (0.04)	0.00 (0.00)	0.43 (0.04)	0
PWIGF	0.47 (0.10)	6905.7	1.69	0.38 (0.12)	0.42 (0.20)^*^	0.79 (0.18)	53
CP8	0.43 (0.03)	12.3	1.96^*^	0.43 (0.03)	0.00 (0.00)	0.43 (0.03)	0
CRIB	0.35 (0.03)	8.2	1.18	0.35 (0.03)	0.00 (0.00)	0.35 (0.03)	0
CIMF	0.35 (0.03)	1.4	−0.35	0.34 (0.03)	0.10 (0.03)^***^	0.44 (0.04)	23
CRBY	0.40 (0.05)	4.2	0.02	0.40 (0.06)	0.18 (0.06)^***^	0.58 (0.07)	31
LLPF	0.29 (0.03)	0.005	1.65	0.29 (0.03)	0.01 (0.03)	0.29 (0.04)	2
SC12	0.68 (0.07)	5.1	2.98^**^	0.62 (0.09)	0.14 (0.14)	0.76 (0.10)	18
PNS24	0.44 (0.08)	502.3	0.91	0.39 (0.12)	0.11 (0.19)	0.50 (0.13)	22
AGECL	0.50 (0.05)	11683.4	−2.76^**^	0.47 (0.05)	0.18 (0.08)^***^	0.65 (0.08)	27
PPAI	0.39 (0.06)	9.5	−0.11	0.39 (0.06)	0.00 (0.00)	0.39 (0.06)	0

ADM = estimates of total phenotype variance (V_P_), t-value of heterozygosity effect (t_He_), proportion of additive genetic variance (V_A_), dominance variance (V_D_) and genetic variance (V_G_) to total phenotype variance (V_P_)1, and ratio of dominance variance to total genetic variance (%)**;** ^1^trait abbreviations are shown in Table 1; ^2^V_P_ is the sum of variance components including error variance in the model; ^*^those that significantly differ from 0 at *P* < 0.05; ^**^those that significantly differ from 0 at *P* < 0.01; and ^***^those that significantly differ from 0 at *P* < 0.001.

The effect of the overall observed heterozygosity (t_He_) for each trait is in Table 3 as a signed t-value. Heterozygosity significantly increased growth traits and SC12 and significantly decreased age at puberty (Table 3). The direction of these effects largely agreed with the preponderance of positive or negative effects at individual SNPs that are reported in Table 2. Dominance variance tended to be significant for traits that have a significant effect of average heterozygosity, such as post-weaning live weight. However, CIMF and CRBY also had significant dominance variance but these traits showed no effect of average heterozygosity. Trait PWIGF had the largest proportion of variance explained by dominance but this estimate had a high standard error due to the small number of records.

### Accuracy of prediction of the phenotypic value

The accuracy of prediction of the phenotypic value using a model that included additive and dominance effects was compared to that obtained with an additive model (Table 4). The accuracy was calculated for the nine traits for which the variance explained by dominance deviations was not equal to 0 (Table 3). The average accuracy across traits was equal to 0.22 for both the additive model and the model with dominance deviations (Table 4). No significant improvement from including dominance deviations was observed for any trait.

**Table 4:** **Average weighted accuracies of predicted phenotypic values across breeds for the 5-fold cross-validation populations based on the GBLUP model without dominance (GRM) and with dominance (GRM + DRM)**

**Trait** ^**1**^	**AA**	**BB**	**BR**	**BX**	**HH**	**MG**	**SG**	**SS**	**TC**	**ALL (SD)**
***GRM***										
PW_hip	0.12	0.33	0.24		0.17		0.16	0.15	0.34	0.275 (0.090)
PW_lwt	0.17	0.28	0.18	0.20	0.14		0.14	0.07	0.29	0.223 (0.072)
X_lwt	0.21	0.20	0.21	0.23	0.22		0.22	0.21	0.33	0.227 (0.043)
PWIGF		0.13							0.07	0.102 (0.047)
CIMF	0.15	0.17	0.27	0.18	0.13	0.21	0.23	0.17	0.24	0.190 (0.046)
CRBY	0.18	0.15	−0.01		−0.04		0.14	0.17		0.117 (0.098)
SC12		0.32								0.318
PNS24		0.23								0.233
AGECL		0.33							0.20	0.260 (0.090)
**Mean**	**0.16**	**0.24**	**0.18**	**0.21**	**0.12**	**0.21**	**0.18**	**0.15**	**0.25**	**0.216 (0.039)**
***GRM + DOM***										
PW_hip	0.13	0.33	0.25		0.16		0.17	0.15	0.34	0.275 (0.088)
PW_lwt	0.16	0.27	0.18	0.23	0.12		0.16	0.06	0.29	0.222 (0.078)
X_lwt	0.21	0.20	0.21	0.25	0.20		0.22	0.21	0.34	0.227 (0.047)
PWIGF		0.15							0.06	0.109 (0.059)
CIMF	0.13	0.15	0.27	0.17	0.13	0.20	0.22	0.15	0.24	0.179 (0.050)
CRBY	0.18	0.17	0.02		−0.03		0.13	0.16		0.121 (0.090)
SC12		0.31								0.314
PNS24		0.23								0.231
AGECL		0.33							0.20	0.257 (0.092)
**Mean**	**0.16**	**0.24**	**0.19**	**0.22**	**0.12**	**0.20**	**0.18**	**0.15**	**0.24**	**0.215 (0.072)**

Cells without values are cases for which they could be not estimated or they were removed when the number of records was less than 200 for the given trait; SD = standard deviation of accuracies across breeds; ^1^trait abbreviations are in Table 1.

### Epistatic effects

**Figure 2:**
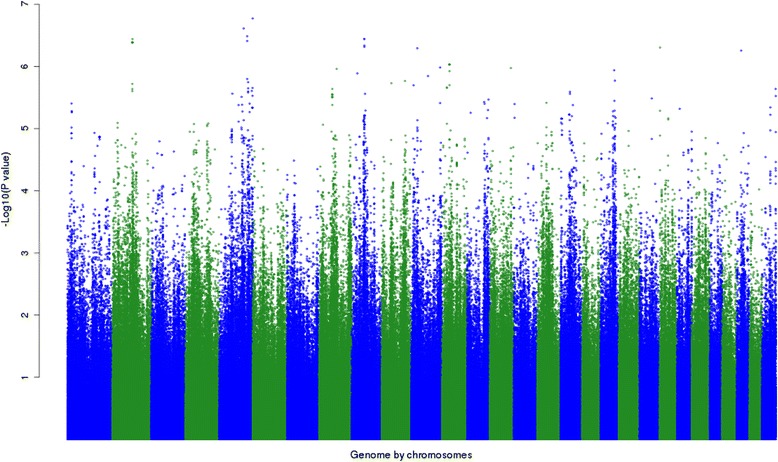
**Manhattan plot showing the –log**_**10**_ **(** ***P*** **-values) of pair-wise epistatic effects for post-weaning live weight (PW_lwt) between lead SNP (BovineHD1400007259 at position 25015640 on BTA 14) and SNPs across the genome, except SNPs on the X chromosome.**

The number of significant (*P* < 10^−5^) epistatic interactions between one lead SNP and one of the other remaining 729 067 SNPs was calculated for the 16 traits (Table 5). For example, for PW_lwt, 153 significant (*P* < 10^−5^) interactions were found between the lead SNP BTA14_25 (close to the *PLAG1* gene) and other SNPs. Two examples of these interactions are in Table 6 and show that the SNPs on chromosome 2 or 5 had a greater effect on weight when the animals carried the A allele at the lead SNP than when they carried the B allele. The minus log *P*-values of epistatic effects for PW_lwt between lead SNP BTA14_25Mb and all other SNPs are shown in Figure 2. Clusters of significant (*P* < 10^−5^) epistatic effects for PW_lwt on BTA2, 5, 8, 9, 19, and 29 were observed (Figure 2). For each cluster, we examined the genes within a region of 50 kb up- and down-stream and, in a few cases, we identified possible candidate genes that could interact with the *PLAG1* gene for PW_lwt. These include the genes *MBNL1* (*muscleblind-like splicing regulator 1*) on BTA1, *FAT/CD36* (*fatty acid translocase/cluster of differentiation 36*) on BTA4, *GRN* (*granulin*), *FASN* (*fatty acid synthase*), and *ITG* (*platelet glycoproteins (A2B and B3)*) on BTA19, and *INS* (*insulin*) and *IGF2* (*insulin-like growth factor 2*) on BTA29.

**Table 5:** **Number of significant epistatic interactions (** ***P*** **< 10** ^**−5**^ **) between each of the 28 lead SNPs and each of the other remaining 729 067 SNPs for each trait**

**Trait** ^**2**^	**Chromosome number and** ***position*** **of the 28 lead SNPs** ^**1**^																											
	**20**	**14**	**5**	**6**	**26**	**25**	**10**	**7**	**29**	**3**	**17**	**13**	**2**	**25**	**6**	**19**	**17**	**8**	**4**	**13**	**12**	**9**	**15**	**23**	**21**	**21**	**9**	**7**
	*49*	*25*	*48*	*40*	*28*	*3.7*	*92*	*99*	*45*	*80*	*25*	*35*	*25*	*15*	*13*	*25*	*61*	*59*	*78*	*66*	*48*	*18*	*58*	*44*	*0.9*	*19*	*101*	*93*
PW_hip	29	24	17	10	4	48	2	25	3	2	3	5	9	39	16	0	34	11	32	6	5	3	1	2	14	3	7	38
X_hip	3	11	22	5	0	14	1	21	24	1	5	9	14	75	7	1	4	20	25	66	2	2	2	7	26	1	0	2
HUMP	0	13	0	201	14	20	6	22	15	0	1	6	0	0	1	8	0	14	28	0	0	24	0	0	1	0	1	1
PW_lwt	39	153	49	3	5	4	18	11	18	10	6	7	7	4	4	18	22	18	17	9	0	6	2	0	26	34	2	8
X_lwt	140	10	33	6	5	389	6	8	8	3	2	13	48	1	31	25	3	4	10	4	17	1	3	5	97	13	9	31
RFI	10	23	9	36	17	19	184	6	28	23	0	12	3	67	5	12	10	20	35	13	0	12	13	0	7	4	25	17
PWIGF	0	28	8	0	7	5	2	12	8	16	3	1	3	2	21	1	1	8	9	12	0	18	4	26	19	14	2	0
CP8	2	14	23	0	4	9	6	3	23	6	6	6	115	6	5	1	0	13	64	7	7	9	2	4	21	2	0	1
CRIB	3	4	31	0	53	8	1	0	42	9	0	3	4	1	0	2	26	36	13	16	11	3	2	1	28	0	109	0
CIMF	57	6	36	0	71	0	2	1	0	15	49	20	3	34	1	41	21	24	0	0	48	6	111	6	13	30	12	113
CRBY	19	9	20	12	11	15	7	0	16	1	0	0	31	0	2	6	9	48	5	1	32	5	0	7	3	4	10	16
LLPF	2	12	41	11	4	28	10	4	27	1	6	6	12	15	29	6	5	32	14	5	3	7	6	7	28	8	11	3
SC12	0	7	0	12	64	28	13	16	10	75	33	23	3	0	23	1	0	75	9	15	0	10	7	0	34	0	6	2
PNS24	0	6	0	14	2	12	12	6	6	11	2	0	10	566	0	5	0	9	5	0	501	23	4	0	29	12	3	0
AGECL	1	13	11	35	1	2	29	3	0	1	4	5	3	4	0	0	3	13	2	83	31	3	0	0	0	1	5	18
PPAI	1	6	4	14	1	26	21	2	1	13	53	20	4	1	19	0	18	3	71	11	0	10	6	2	58	0	196	1

^1^Numbers in bold are chromosome numbers and numbers in italics are positions in Mb; ^2^trait abbreviations are in Table 1.

**Table 6:** **Example of significant epistasis of the lead SNP BTA14_25Mb with SNPs BTA2_70715761 and BTA29_50068561) for post-weaning live weight (PW_lwt, kg)**

	**Lead SNP**	
	**A allele**	**B allele**
BTA2_70715761		
A allele	0	3.732
B allele	−6.769	1.023^*^
BTA5_103576732		
A allele	0	3.25
B allele	−4.162	2.954^*^

^*^This was calculated as the sum of the effect of B allele of the lead SNP and the effect of the SNP and their interaction.

Analysis of epistatic effects shows that the number of significant interactions between SNPs was greater than expected by chance and varied widely between traits (Table 5). As described in Bolormaa et al. [18], the first four lead SNPs have an allele that increased mature size (increases height and weight and decreased fatness, RFI and blood concentration of IGF1). The highest number of epistatic effects between these lead SNPs and other SNPs was found for weight (PW_lwt; X_lwt), height (HUMP; PW_hip), fatness (CIMF; CRIB), RFI and PWIGF. In addition, large numbers of significant interactions with other SNPs were found for lead SNPs 5_48Mb and 6_40Mb for LLPF and AGECL, respectively.

Five lead SNPs (BTA7_98Mb; BTA10_92Mb; BTA25_3.7 Mb; BTA26_28Mb; BTA29_45Mb) had an allele that increased meat tenderness and fatness, as described by Bolormaa et al. [18] and showed interactions that had an effect on weight (X_lwt), RFI, fatness and SC12 (Table 5). The next seven lead SNPs (BTA2_25Mb; BTA3_80Mb; BTA6_13Mb; BTA13_35Mb; BTA17_25; BTA19_25Mb; BTA25_15Mb) had an allele that increased both fatness and weight [18] and showed interactions that affected RFI, CIMF and SC12.

## Discussion

Traits related to fitness, including growth rate, are commonly found to show inbreeding depression and heterosis [30,31], which is usually explained by directional dominance at loci that control these traits. Our results are consistent with this. We found an effect of average heterozygosity on traits related to fitness, such that increasing heterozygosity changed the trait in the direction that is presumed to increase fitness, for instance, increased growth rate. In accordance with this, more individual SNPs had dominance effects in the direction presumed to increase fitness than in the reverse direction. However, the FDR associated with these effects was moderate to high, so the evidence for significant dominance effects at individual SNPs was not strong, especially for traits that do not have a clear relationship to fitness, such as fat depth, retail beef yield and tenderness. The FDR of dominance effects (ranging from 26.1 to 109.9%; Table 2) was considerably higher than the FDR of additive effects in the same data (ranging from 1.5 to 21.1%; [15,16]). This suggests that dominance effects are smaller than additive effects and/or more difficult to estimate (note that an estimated FDR greater than 100% occurs when the number of significant SNPs is smaller than expected by chance).

Directional dominance can cause a significant regression of trait phenotype on average heterozygosity, as we observed. In fact, the effect of average heterozygosity on trait phenotype could be considered as an estimate of inbreeding depression based on SNP genotypes. However, significant inbreeding depression can occur without generating much dominance variance if it is due to small dominance deviations at very many loci. The fact that we observed significant dominance variance for some traits implies that some individual loci have moderate dominance effects. This is supported by the finding of significant dominance effects at individual SNPs in the GWAS.

Across traits, the proportion of phenotypic variance that was estimated to be due to dominance varied widely (from 0 to 42%) but much of this variation could be due to large standard errors of the estimates. The median values across traits for the dominance variance as a proportion of total phenotypic variance is 0.05 compared with a median value for the additive variance which was 0.4 of the total phenotypic variance. The results for post-partum anoestrous interval (PPAI) are somewhat surprising for a fertility trait for the following reasons: (1) the heritability for PPAI was equal to 0.39, which is higher than many heritability estimates of fertility traits in beef cattle [32]; (2) there was weak evidence at best for dominance effects of individual SNPs; (3) there was no significant effect of average heterozygosity and (4) the estimate of dominance variance was close to zero. Fertility traits are usually considered to be fitness traits and the expectation is therefore that non-additive effects are important. The results reported here may indicate lack of power in this subset of the data but it could also be that an intermediate value of PPAI is optimal for fitness.

The model that we used to estimate dominance variance is similar to that of Vitezica et al. [29], except that SNPs were weighted differently to calculate the DRM and we also fitted the average heterozygosity. Variation in average heterozygosity is equivalent to variation in inbreeding in a classical model and thus, we accounted for the effect of this on the phenotype. Our assumptions that E(d) = 0 and cov(a,d) = 0 hold after correcting for average heterozygosity (inbreeding). If average heterozygosity is not included in the model, then the average effects of inbreeding contribute to the estimate of dominance variance. The usual definition of dominance variance (e.g., [33]) does not include the effect of variation in inbreeding and we have followed that practice.

There are a few publications (e.g., [3-5,34]) that have reported estimates of the non-additive genetic variance based on pedigree information in cattle. Using an animal model, Rodriguez-Almeida et al. [34] estimated the dominance variance at 28% and 11% of total phenotypic variance for weaning weight and hip height, respectively, while we obtained lower estimates for weaning live weight (11%) and a hip height (4%). In dairy cattle, estimates of dominance variance for milk, fat, and protein yields have been reported by many authors [3,35-37], with values ranging from 3 to 24% of total phenotypic variance. Palucci et al. [4] estimated the dominance variance for fertility traits such as non-return rate (0.6% of total phenotypic variance) and calving to first service (7.3% of total phenotypic variance) in Canadian dairy cows. Our estimates of dominance variance as a proportion of total genetic variance were also low for fertility traits, but it should be noted that there was insufficient power due to the limited number of records for these traits.

Su et al. [6] estimated non-additive genetic variance for average daily gain in pigs using high-density SNPs and reported that 5.6 and 9.5% of total phenotypic variance was explained by dominance and additive by additive variance, respectively, which is similar to our median estimate of 5%. However, they did not use the classical definition of dominance variance and consequently their dominance variance might be slightly over-estimated.

We used a set of animals that originated from multiple breeds. The extension of the traditional model with additive and dominance variance components to multiple breeds has been done in two ways. Either the breeds are regarded as separate populations (e.g., [38]) or the breeds are regarded as partially inbred lines within a larger population [33]. Both formulations result in the definition of multiple variance components. For instance, the model with inbreeding [33] has five variance components, as well as the regression on average heterozygosity. Consequently, it is not possible to obtain accurate estimates of all parameters using real data and thus, these models are seldom used. The model that we used considered partially inbred lines within a larger population but the variances were defined in terms of SNP genotype effects. The model is best understood from a model that is equivalent to model (2), in which the breeding value (**g**) and the dominance value (**d**) are replaced by the sum of all the effects of individual SNPs, that is **g = Tu** and **d = Hv**. Thus, our model can be considered as estimating the combined variances due to all SNPs. **T** and **H** are defined using the allele frequencies over the whole dataset and thus correspond to a model in which the current breeds have descended from a base population with these allele frequencies, and the current breeds are regarded as inbred relative to this base population. Consequently, the diagonal elements of the GRM and DRM are not expected to be 1. Diagonal elements of the GRM are greater than 1 when calculated by **TT’**/m, as expected. Diagonal elements of the DRM calculated from **HH’**/m are also typically greater than 1, which reflects the effect of inbreeding. The estimated variance components refer to the variances in the hypothetical base population. A potential problem is that the additive (T) and dominance (H) values are not necessarily orthogonal when genotype frequencies deviate from Hardy-Weinberg equilibrium. However, the results in Table 3 show that the additive variances obtained with the AM and ADM models were almost the same. Therefore, additive and dominance effects were nearly orthogonal.

Although there were more significant epistatic effects than expected by chance, the FDR was high (overall FDR of 39% at *P* < 10^−5^). This could be due to the additive × additive epistatic variance being small and/or to the epistatic effects being difficult to estimate. There were no significant epistatic interactions among the 28 lead SNPs, for which the power for detecting such interactions was expected to be greatest, since the lead SNPs had highly significant additive effects on multiple traits. By focusing on these 28 SNPs, we may have missed many important epistatic interactions. However, testing interactions between all pairs of SNPs would have greatly increased the multiple-testing problem. Therefore, caution is warranted in interpreting the significant interactions that we identified. Still, an interaction between *PLAG1* and *IGF2* is biologically plausible, since PLAG1 is a transcription factor that regulates many genes and pathways, including the *IGF2* and *IGF1R* pathways [39].

To the best of knowledge, there are no publications on the estimation of non-additive genetic variance in beef cattle using high-density SNPs. Only a few papers [6,34] have reported results on the variance explained by epistatic (additive by additive) variance in dairy cattle and pigs. Su et al. [6] used a Hadamard product of GRM × GRM to calculate the epistatic relationship matrix. Since the off-diagonal elements of the GRM in our data were small, the square of these numbers will be close to 0, which means that the estimation of epistatic variance would suffer from a large sampling error and therefore we did not attempt it.

Although the dominance variance was significant for some traits, inclusion of dominance deviations in genomic prediction did not increase the accuracy of prediction of the genetic value of individuals and hence the prediction of their phenotypic values. This is consistent with the high FDR of dominance effects, which indicates that the dominance effects were not estimated with high precision. This could be because the dominance effects were small and/or because they were difficult to estimate. For instance, all three genotypes at a given SNP must be present in the data to estimate the dominance effect. Su et al. [6] reported an increase in accuracy of prediction (from 0.319 to 0.330) as a result of fitting a dominance effect in pigs but their data included pigs that were more closely related to each other than the animals in our dataset. Therefore, inclusion of dominance effects in genetic evaluations for important traits (e.g., CIMF and AGECL) could increase the accuracy of prediction of genetic effects, but only if the animals are closely related. In these situations, caution should be taken to minimize the tendency to confound dominance with common environment effects such as litter.

## Conclusions

Across all SNPs, we observed dominance effects for growth and fertility traits in the direction that is expected to increase fitness. The dominance effects were not equal for all SNPs, which resulted in some dominance effects reaching significance (*P* < 10^−5^) and significant variance explained by dominance deviations for some traits. Despite this, including dominance in the prediction model did not increase the accuracy of prediction of genetic and phenotypic values for any trait, which is probably because the dominance variance is much smaller than the additive genetic variance, and dominance effects are difficult to estimate accurately. The number of additive × additive epistatic effects was greater than expected by chance but their FDR was high.
